# 4,4,5,5-Tetra­methyl-2-(4-pyridinio)-2-imidazoline-1-oxyl-3-oxide perchlorate

**DOI:** 10.1107/S1600536809013531

**Published:** 2009-04-18

**Authors:** Zhi-Yong Gao, Jiu-Li Chang, Dong Xian, Kai Jiang

**Affiliations:** a College of Chemistry and Environmental Science, Henan Normal University, Xinxiang 453002, People’s Republic of China

## Abstract

The crystal structure of the title compound, C_12_H_17_N_3_O_2_^+^·ClO_4_^−^, consists of 4,4,5,5-tetra­methyl-2-(4-pyridinio)imidazoline-1-oxyl-3-oxide radical cations and perchlorate anions. Both the cation and the Cl atom of the anion are located on the same twofold rotation axis, and the crystal structure shows the average structure for the radical cation. The five-membered ring assumes a half-chair conformation. The cation links with the anion *via* N—H⋯O hydrogen bonding.

## Related literature

For general background, see: Wang *et al.* (2004 ▶); Li *et al.* (2003 ▶); Kahn *et al.* (2000 ▶); Tsukahara *et al.* (2003 ▶); Fettouhi *et al.* (2003 ▶); Zhang *et al.* (2004 ▶); Fokin *et al.* (2004 ▶); Chang *et al.* (2009 ▶). For the synthesis, see: Ullman *et al.* (1970 ▶, 1972 ▶).
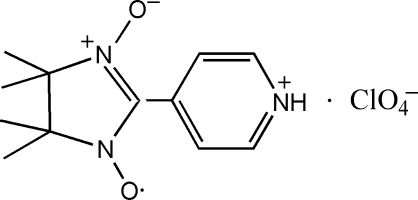


## Experimental

### 

#### Crystal data

C_12_H_17_N_3_O_2_^+^·ClO_4_^−^
*M* *_r_* = 334.74
Orthorhombic, 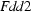

*a* = 17.485 (4) Å
*b* = 11.854 (2) Å
*c* = 14.921 (2) Å
*V* = 3092.6 (10) Å^3^
*Z* = 8
Mo *K*α radiation
μ = 0.28 mm^−1^
*T* = 273 K
0.33 × 0.26 × 0.23 mm

#### Data collection

Bruker SMART CCD area-detector diffractometer
Absorption correction: multi-scan (*SADABS*; Sheldrick, 1996 ▶) *T*_min_ = 0.915, *T*_max_ = 0.930
3994 measured reflections
1204 independent reflections
1169 reflections with *I* > 2σ(*I*)
*R*_int_ = 0.016

#### Refinement

*R*[*F* ^2^ > 2σ(*F* ^2^)] = 0.033
*wR*(*F* ^2^) = 0.102
*S* = 1.06
1204 reflections
103 parameters
1 restraint
H-atom parameters constrained
Δρ_max_ = 0.27 e Å^−3^
Δρ_min_ = −0.15 e Å^−3^
Absolute structure: Flack (1983 ▶), 457 Friedel pairs
Flack parameter: 0.12 (10)

### 

Data collection: *SMART* (Bruker, 2002 ▶); cell refinement: *SAINT* (Bruker, 2002 ▶); data reduction: *SAINT*; program(s) used to solve structure: *SHELXTL* (Sheldrick, 2008 ▶); program(s) used to refine structure: *SHELXTL*; molecular graphics: *SHELXTL*; software used to prepare material for publication: *SHELXTL*.

## Supplementary Material

Crystal structure: contains datablocks I, global. DOI: 10.1107/S1600536809013531/xu2504sup1.cif

Structure factors: contains datablocks I. DOI: 10.1107/S1600536809013531/xu2504Isup2.hkl

Additional supplementary materials:  crystallographic information; 3D view; checkCIF report

## Figures and Tables

**Table 1:** Hydrogen-bond geometry (Å, °)

*D*—H⋯*A*	*D*—H	H⋯*A*	*D*⋯*A*	*D*—H⋯*A*
N1—H1*B*⋯O3	0.86	2.20	2.963 (4)	149

**Fig. 1.**
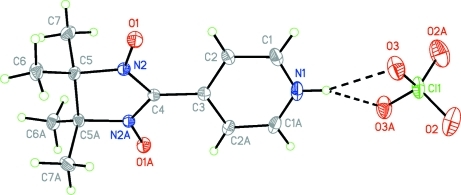
The molecular structure of the title compound with atom labeling. The thermal ellipsoids are drawn at 30% probability level [symmetry code: (A) -x,-y+1,z]. Dashed lines indicate hydrogen bonding.

## supplementary crystallographic information

### Comment

The major research aims in the field of molecular magnetism are on one hand the chemical design of molecular assemblies that exhibit a spontaneous magnetization and on the other hand the rationalization of magneto-structural correlation (Wang *et al.*, 2004; Li *et al.*, 2003; Kahn *et al.*, 2000; Tsukahara *et al.*, 2003). Nitronyl nitroxide radicals (NITR), stable organic radicals, have played an important role in the design and synthesis of molecular magnetic materials (Fettouhi *et al.*, 2003; Zhang *et al.*, 2004; Fokin *et al.*, 2004). Many structures have been investigated on the coordination of nitronyl nitroxide radicals to metals, but less on the non-covalent weak interactions of nitronyl nitroxide radicals with other molecules (Chang *et al.*, 2009). Taking account of these, we report on the molecular assemblies of NITpPy and perchlorate anion in order to further understand the coordination chemistry of nitronyl nitroxide radicals.

The structure of the title compound is shown in Fig. 1. The compound consists of a discrete [NITpPyH] cation and a perchlorate anion. NITpPy acts as a proton sponge by accepting a proton. The transfer of protons results in symmetric intermolecular hydrogen bonds: the double hydrogen bonds occur between two oxygen atoms from perchlorate anion and one nitrogen atom from the pyridyl ring (Table 1). The nitronyl nitroxide fragment O—N—C—N—O is almost coplanar, but make a dihedral angle of 17.0° with the pyridyl ring. In the unit cell cations and anions are alternatively arranged.

### Experimental

NITpPy was synthesized according to a literature procedure (Ullman *et al.*, 1970; Ullman *et al.*, 1972). The title compound was obtained serendipitously from the reaction of copper perchlorate hydrate (1 mmol) and NITpPy (2 mmol) in methanol (10 ml). The mixture was stirred for 4 h at room temperature and then filtered. Subsequently, the filtrate was diffused with diethyl ether vapor and dark-purple block crystals were obtained one week later.

### Refinement

The H atoms were positioned geometrically and refined using the riding-model approximation, with C—H = 0.93 (aromatic), 0.96 Å (methyl) and N—H = 0.86 Å, and U_iso_(H) = 1.5U_eq_(C) for methyl groups and 1.2U_eq_(C,N) for others.

### Crystal data

**Table:**

C_12_H_17_N_3_O_2_^+^·ClO_4_^−^	*F*(000) = 1400
*M**_r_* = 334.74	*D*_x_ = 1.438 Mg m^−^^3^
Orthorhombic, *F**d**d*2	Mo *K*α radiation, λ = 0.71073 Å
Hall symbol: F 2 -2d	Cell parameters from 2949 reflections
*a* = 17.485 (4) Å	θ = 2.3–29.0°
*b* = 11.854 (2) Å	µ = 0.28 mm^−^^1^
*c* = 14.921 (2) Å	*T* = 273 K
*V* = 3092.6 (10) Å^3^	Block, dark-purple
*Z* = 8	0.33 × 0.26 × 0.23 mm

### Data collection

**Table:**

Bruker SMART CCD area-detector diffractometer	1204 independent reflections
Radiation source: fine-focus sealed tube	1169 reflections with *I* > 2σ(*I*)
graphite	*R*_int_ = 0.016
φ and ω scans	θ_max_ = 25.5°, θ_min_ = 2.5°
Absorption correction: multi-scan (*SADABS*; Sheldrick, 1996)	*h* = −21→20
*T*_min_ = 0.915, *T*_max_ = 0.930	*k* = −11→14
3994 measured reflections	*l* = −15→18

### Refinement

**Table:**

Refinement on *F*^2^	Hydrogen site location: inferred from neighbouring sites
Least-squares matrix: full	H-atom parameters constrained
*R*[*F*^2^ > 2σ(*F*^2^)] = 0.033	*w* = 1/[σ^2^(*F*_o_^2^) + (0.0762*P*)^2^ + 1.0334*P*] where *P* = (*F*_o_^2^ + 2*F*_c_^2^)/3
*wR*(*F*^2^) = 0.102	(Δ/σ)_max_ < 0.001
*S* = 1.06	Δρ_max_ = 0.27 e Å^−^^3^
1204 reflections	Δρ_min_ = −0.15 e Å^−^^3^
103 parameters	Extinction correction: *SHELXTL* (Bruker, 2000), Fc^*^=kFc[1+0.001xFc^2^λ^3^/sin(2θ)]^-1/4^
1 restraint	Extinction coefficient: 0.0026 (5)
Primary atom site location: structure-invariant direct methods	Absolute structure: Flack (1983), 457 Friedel pairs
Secondary atom site location: difference Fourier map	Flack parameter: 0.12 (10)

### Special details

**Table:**

Geometry. All e.s.d.'s (except the e.s.d. in the dihedral angle between two l.s. planes) are estimated using the full covariance matrix. The cell e.s.d.'s are taken into account individually in the estimation of e.s.d.'s in distances, angles and torsion angles; correlations between e.s.d.'s in cell parameters are only used when they are defined by crystal symmetry. An approximate (isotropic) treatment of cell e.s.d.'s is used for estimating e.s.d.'s involving l.s. planes.
Refinement. Refinement of *F*^2^ against ALL reflections. The weighted *R*-factor *wR* and goodness of fit *S* are based on *F*^2^, conventional *R*-factors *R* are based on *F*, with *F* set to zero for negative *F*^2^. The threshold expression of *F*^2^ > σ(*F*^2^) is used only for calculating *R*-factors(gt) *etc*. and is not relevant to the choice of reflections for refinement. *R*-factors based on *F*^2^ are statistically about twice as large as those based on *F*, and *R*- factors based on ALL data will be even larger.

### Fractional atomic coordinates and isotropic or equivalent isotropic displacement parameters (Å^2^)

**Table:**

	*x*	*y*	*z*	*U*_iso_*/*U*_eq_	
Cl1	0.0000	0.5000	0.05797 (7)	0.0787 (4)	
O2	0.0203 (2)	0.4065 (5)	0.0039 (3)	0.1497 (15)	
O3	0.06280 (15)	0.5281 (3)	0.1140 (2)	0.1055 (9)	
N1	0.0000	0.5000	0.2971 (2)	0.0684 (10)	
H1B	0.0000	0.5000	0.2395	0.082*	
N2	0.05399 (9)	0.54721 (12)	0.62987 (12)	0.0399 (4)	
C1	0.04640 (17)	0.5698 (2)	0.33929 (18)	0.0626 (6)	
H1A	0.0785	0.6169	0.3067	0.075*	
C2	0.04743 (13)	0.57302 (19)	0.43134 (16)	0.0520 (5)	
H2A	0.0793	0.6232	0.4614	0.062*	
C3	0.0000	0.5000	0.4789 (2)	0.0417 (6)	
C4	0.0000	0.5000	0.5764 (2)	0.0379 (6)	
C5	0.02991 (11)	0.54883 (16)	0.72622 (14)	0.0425 (5)	
C6	0.09853 (14)	0.5301 (2)	0.78664 (19)	0.0630 (6)	
H6A	0.1322	0.5939	0.7827	0.095*	
H6B	0.1253	0.4634	0.7680	0.095*	
H6C	0.0816	0.5213	0.8474	0.095*	
C7	−0.00457 (14)	0.66478 (19)	0.74312 (19)	0.0616 (7)	
H7A	0.0352	0.7206	0.7422	0.092*	
H7B	−0.0293	0.6654	0.8006	0.092*	
H7C	−0.0414	0.6815	0.6972	0.092*	
O1	0.11652 (8)	0.59302 (15)	0.60418 (12)	0.0599 (5)	

### Atomic displacement parameters (Å^2^)

**Table:**

	*U*^11^	*U*^22^	*U*^33^	*U*^12^	*U*^13^	*U*^23^
Cl1	0.0631 (5)	0.1338 (9)	0.0392 (5)	−0.0116 (5)	0.000	0.000
O2	0.154 (3)	0.199 (4)	0.096 (3)	0.023 (3)	0.013 (2)	−0.053 (3)
O3	0.0789 (14)	0.167 (2)	0.0707 (17)	−0.0372 (15)	−0.0088 (13)	0.0105 (16)
N1	0.092 (2)	0.0776 (18)	0.0360 (17)	0.0404 (17)	0.000	0.000
N2	0.0356 (7)	0.0440 (7)	0.0400 (10)	−0.0075 (6)	−0.0005 (7)	−0.0002 (6)
C1	0.0729 (15)	0.0692 (14)	0.0457 (14)	0.0203 (11)	0.0104 (11)	0.0154 (11)
C2	0.0547 (12)	0.0570 (10)	0.0443 (13)	0.0068 (9)	0.0035 (9)	0.0088 (9)
C3	0.0394 (13)	0.0430 (12)	0.0425 (18)	0.0116 (10)	0.000	0.000
C4	0.0367 (12)	0.0376 (12)	0.0394 (18)	−0.0002 (9)	0.000	0.000
C5	0.0409 (10)	0.0499 (10)	0.0368 (11)	−0.0056 (9)	−0.0015 (9)	−0.0025 (8)
C6	0.0548 (12)	0.0865 (15)	0.0478 (15)	−0.0160 (12)	−0.0141 (11)	0.0046 (11)
C7	0.0683 (14)	0.0550 (11)	0.0614 (17)	−0.0014 (10)	0.0048 (12)	−0.0153 (10)
O1	0.0461 (7)	0.0784 (10)	0.0553 (11)	−0.0250 (7)	0.0047 (7)	0.0008 (8)

### Geometric parameters (Å, °)

**Table:**

Cl1—O2^i^	1.416 (4)	C2—H2A	0.9300
Cl1—O2	1.416 (4)	C3—C2^i^	1.393 (3)
Cl1—O3	1.420 (3)	C3—C4	1.455 (4)
Cl1—O3^i^	1.420 (3)	C4—N2^i^	1.357 (2)
N1—C1	1.318 (4)	C5—C6	1.517 (3)
N1—C1^i^	1.318 (4)	C5—C7	1.522 (3)
N1—H1B	0.8600	C5—C5^i^	1.560 (4)
N2—O1	1.280 (2)	C6—H6A	0.9600
N2—C4	1.357 (2)	C6—H6B	0.9600
N2—C5	1.498 (3)	C6—H6C	0.9600
C1—C2	1.374 (4)	C7—H7A	0.9600
C1—H1A	0.9300	C7—H7B	0.9600
C2—C3	1.393 (3)	C7—H7C	0.9600
			
O2^i^—Cl1—O2	110.5 (5)	N2—C4—N2^i^	108.0 (3)
O2^i^—Cl1—O3	110.2 (3)	N2—C4—C3	126.01 (13)
O2—Cl1—O3	109.0 (2)	N2^i^—C4—C3	126.01 (13)
O2^i^—Cl1—O3^i^	109.0 (2)	N2—C5—C6	110.26 (18)
O2—Cl1—O3^i^	110.2 (3)	N2—C5—C7	106.37 (18)
O3—Cl1—O3^i^	107.9 (2)	C6—C5—C7	110.28 (19)
C1—N1—C1^i^	123.0 (3)	N2—C5—C5^i^	100.31 (10)
C1—N1—H1B	118.5	C6—C5—C5^i^	114.96 (17)
C1^i^—N1—H1B	118.5	C7—C5—C5^i^	113.9 (2)
O1—N2—C4	126.40 (18)	C5—C6—H6A	109.5
O1—N2—C5	121.47 (16)	C5—C6—H6B	109.5
C4—N2—C5	111.96 (16)	H6A—C6—H6B	109.5
N1—C1—C2	120.2 (3)	C5—C6—H6C	109.5
N1—C1—H1A	119.9	H6A—C6—H6C	109.5
C2—C1—H1A	119.9	H6B—C6—H6C	109.5
C1—C2—C3	118.9 (2)	C5—C7—H7A	109.5
C1—C2—H2A	120.5	C5—C7—H7B	109.5
C3—C2—H2A	120.5	H7A—C7—H7B	109.5
C2^i^—C3—C2	118.8 (3)	C5—C7—H7C	109.5
C2^i^—C3—C4	120.61 (16)	H7A—C7—H7C	109.5
C2—C3—C4	120.61 (16)	H7B—C7—H7C	109.5
			
C1^i^—N1—C1—C2	−0.68 (16)	C2—C3—C4—N2	−15.56 (13)
N1—C1—C2—C3	1.3 (3)	C2^i^—C3—C4—N2^i^	−15.56 (13)
C1—C2—C3—C2^i^	−0.65 (15)	C2—C3—C4—N2^i^	164.44 (13)
C1—C2—C3—C4	179.35 (15)	O1—N2—C5—C6	−39.6 (2)
O1—N2—C4—N2^i^	175.0 (2)	C4—N2—C5—C6	144.69 (16)
C5—N2—C4—N2^i^	−9.58 (9)	O1—N2—C5—C7	80.0 (2)
O1—N2—C4—C3	−5.0 (2)	C4—N2—C5—C7	−95.74 (16)
C5—N2—C4—C3	170.42 (9)	O1—N2—C5—C5^i^	−161.21 (18)
C2^i^—C3—C4—N2	164.44 (13)	C4—N2—C5—C5^i^	23.1 (2)

Symmetry codes: (i) −*x*, −*y*+1, *z*.

### Hydrogen-bond geometry (Å, °)

**Table:**

*D*—H···*A*	*D*—H	H···*A*	*D*···*A*	*D*—H···*A*
N1—H1B···O3	0.86	2.20	2.963 (4)	149
